# Case Report: Radiation necrosis mimicking tumor progression in a patient with extranodal natural killer/T-cell lymphoma

**DOI:** 10.3389/fradi.2023.1257565

**Published:** 2023-10-25

**Authors:** Boxiao Chen, Yili Fan, Luyao Wang, Jiawei Zhang, Dijia Xin, Xi Qiu, Huawei Jiang, Baizhou Li, Qin Chen, Chao Wang, Xibin Xiao, Liansheng Huang, Yang Xu

**Affiliations:** ^1^ Department of Hematology, The Second Affiliated Hospital, Zhejiang University School of Medicine, Hangzhou, China; ^2^ Department of Pathology, The Second Affiliated Hospital, Zhejiang University School of Medicine, Hangzhou, China; ^3^ Department of Radiology, The Second Affiliated Hospital, Zhejiang University School of Medicine, Hangzhou, China; ^4^ Zhejiang Provincial Key Laboratory for Cancer Molecular Cell Biology, Life Sciences Institute, Zhejiang University, Hangzhou, China

**Keywords:** radiation necrosis, secondary central nervous system lymphoma, extranodal natural killer/T-cell lymphoma, radiation therapy, radiological examination

## Abstract

Radiation-induced cerebral necrosis, also known as radiation encephalopathy, is a debilitating condition that significantly impacts the quality of life for affected patients. Secondary central nervous system lymphoma (SCNSL) typically arises from highly aggressive mature B-cell lymphoma, but rarely from extranodal natural killer T-cell lymphoma (ENKTL). Treatment will be guided by differentiation between lymphoma progression from brain necrosis, and is particularly important for critically ill patients in an acute setting. However, differential diagnosis remains challenging because they share similar clinical manifestations and have no specific imaging features. We present the case of a 52-year-old man with ENKTL who suffered an emergency brain herniation secondary to massive radiation necrosis. The diagnosis established by brain biopsy ultimately led to appropriate treatment. The importance of the diagnostic biopsy is highlighted in this case for distinguishing between radiation necrosis and SCNSL.

## 1. Introduction

Extranodal natural killer/T-cell lymphoma, nasal type represents a rare subtype of Epstein-Barr virus (EBV)-related lymphoma, which occurs more frequently in Asian and South America than in North America and Europe (1). ENKTL initially arises in the nasal cavity, nasopharynx and paranasal sinuses, and may disseminate to the gastrointestinal tract, skin, testes, eyes or soft tissues; less than 6% of ENKTL involves central nervous system (CNS), where lymphoma infiltrates or compresses the brain parenchyma, spinal cord and leptomeninges, resulting in a variety of neurological symptoms (2). The concurrent CNS involvement or CNS relapse of ENKTL is associated with poor prognosis due to the lack of optimal prophylaxis and treatment.

Radiation therapy (RT), given in concurrent, sequential or sandwich chemoradiation, has an essential role in improved overall survival (OS) and disease-free survival (DFS), especially in patients with localized ENKTL. Involved-site RT (ISRT) is recommended as the appropriate modality to minimize radiation-induced toxicity. However, radiation can cause subacute damages to neuroglial cells as well as intracranial vasculature, resulting in radiation necrosis, a delayed complication characterized by late-onset neurocognitive dysfunction, such as diminished mental capacity for language competency, learning ability, interpersonal communication, and intelligence (3). In most cases, radiation necrosis usually appears insidiously but progresses rapidly at the late stage, and becomes indistinguishable from CNS involvement by ENTKL during follow-up.

Here we present a clinical ENTKL case of a patient who suffered radiation necrosis mimicking lymphoma progression, report clinical, imaging and pathological findings, and discuss the diagnostic and therapeutic options.

## 2. Case presentation

**Figure 1:**
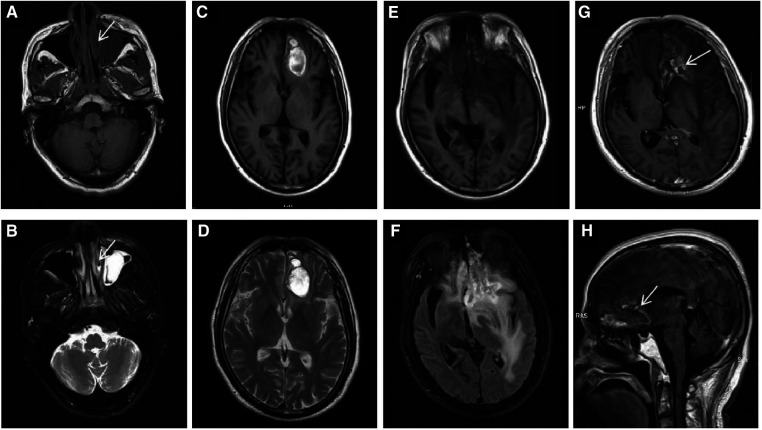
In October 2019, an axial paranasal sinus MRI on first presentation (**A,B**, arrows) demonstrated mucosal swelling of the left nasal cavity with low signal on T1WI and high signal on T2WI. The mucosa of bilateral maxillary sinus, ethmoid sinus and left frontal sinus were thickened, especially the left maxillary sinus with a soft tissue mass, which were considered to be inflammation. In May 2020, brain MRI (**C,D**) revealed abnormal signal in the left frontal lobe showing mixed signal on T1WI and heterogeneous high signal on T2WI, causing mild peritumoral edema, mild compression of the anterior horn of the left ventricle and slight midline shift. Consequently, lymphoma involvement with hemorrhage was considered. In November 2021, preoperative brain MRI (**E,F**) showed an irregular mass in the left frontal lobe, with mixed intensity signal on T1WI and heterogeneous high signal on T2-FLAIR. After enhancement, axial (**G**, arrow) and sagittal (**H**, arrow) brain MRI showed the mass was wreath-like enhancement. The left anterior horn of the ventricle was compressed, the midline structure was obviously shifted to the right, and the left optic tract and anterior commissure were compressed. The cisterna of ventricles enlarged and the cerebral sulci deepened.

**Figure 2:**
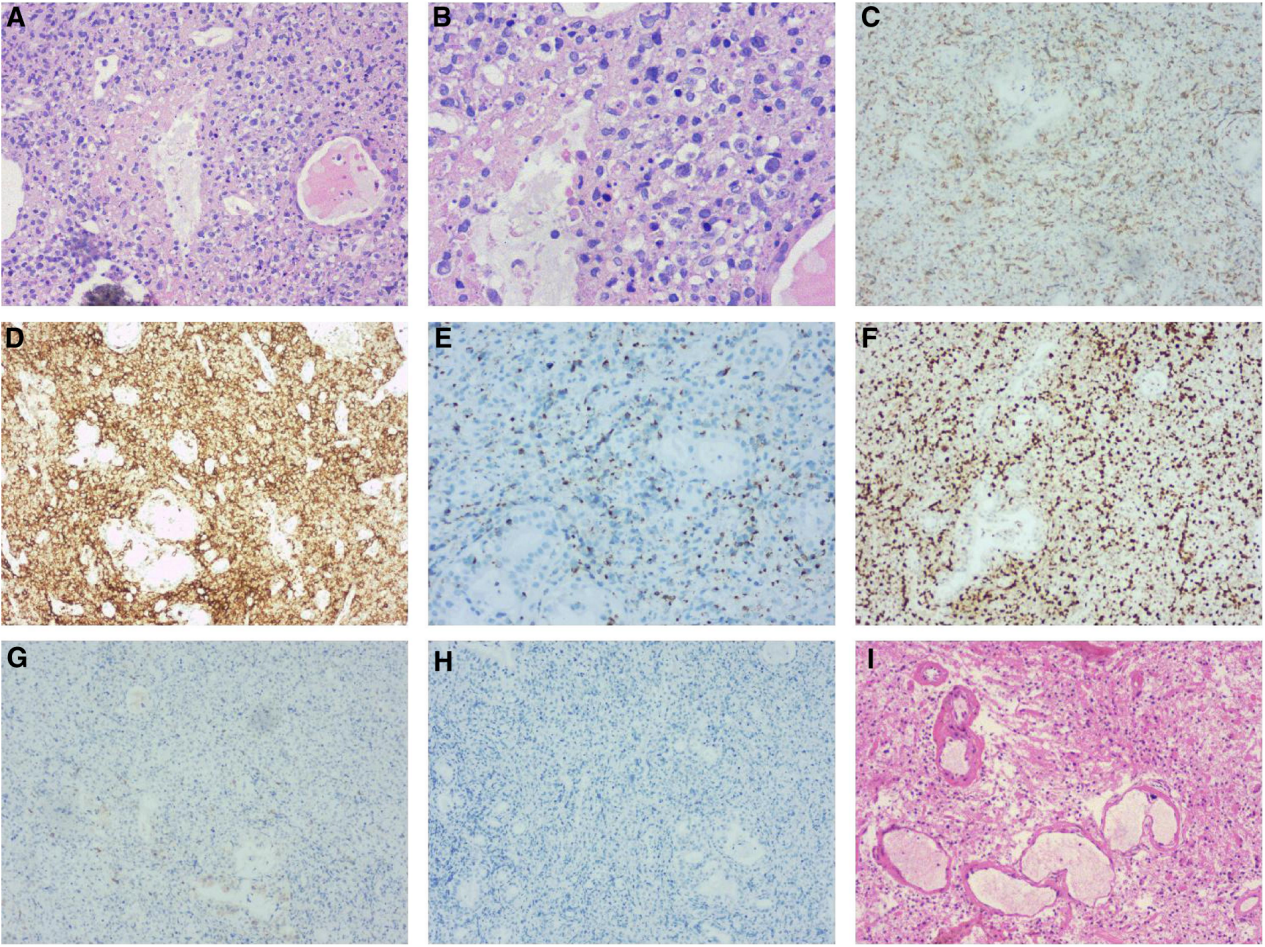
Left nasal cavity biopsy (**A,B**) demonstrated diffuse infiltration of atypical cells with coarse chromatin, prominent nucleoli by H&E-stain. Immunohistochemical stains (C to H) revealed the tumor cells expressing cytoplasmic CD3 (**C**), CD30, CD56 (**D**), TIA-1 (**E**) and MUM-1 were positive with Ki-67 (**F**) index of >40%, but negative for the rest of the T-cell markers including CD5 (**G**) and ALK/P80 (**H**) Post-operative pathology (**I**) showed necrosis of the left frontal brain tissue and hyperplasia of histocytes without neoplastic cells.

**Figure 3:**
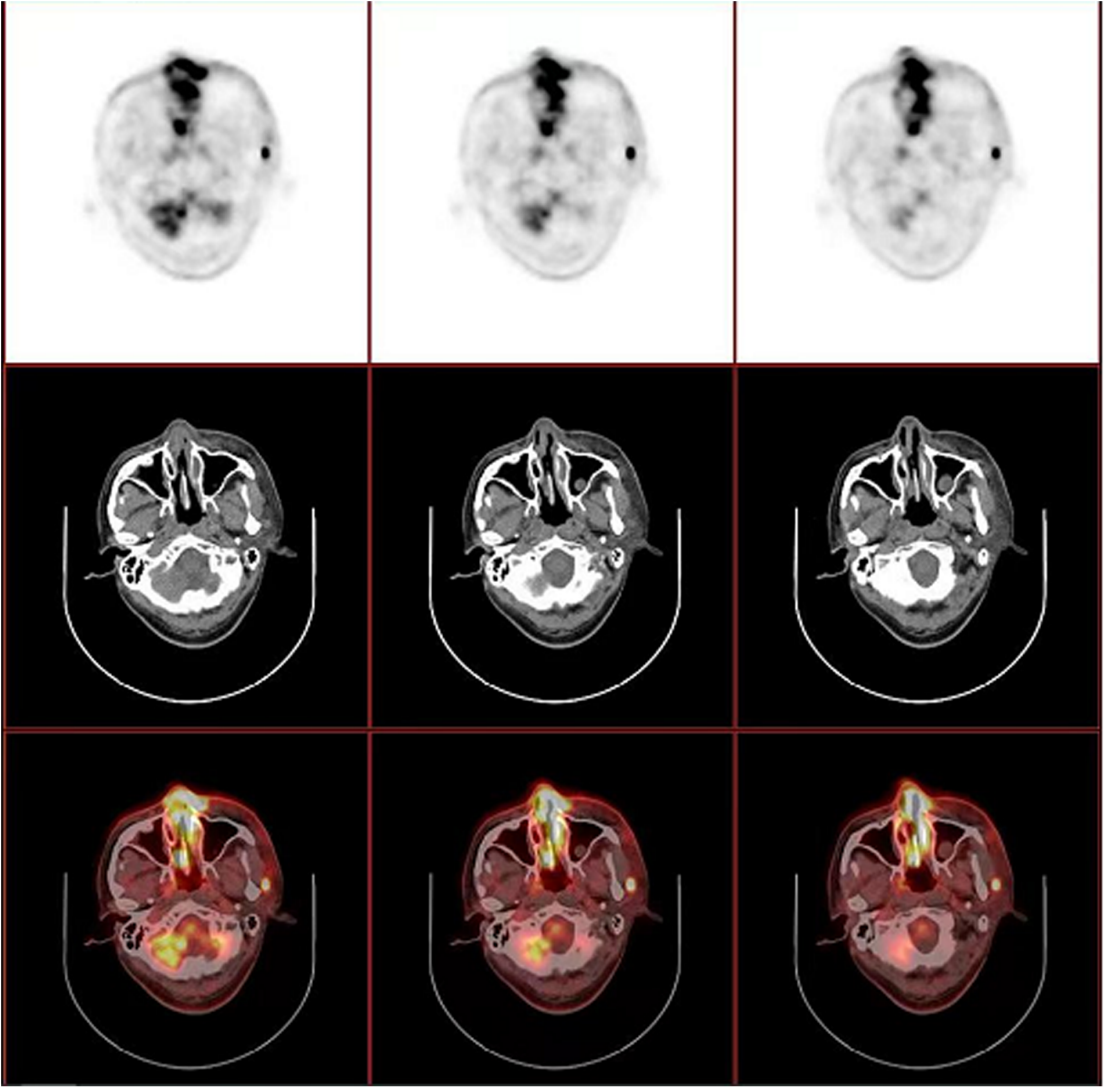
PET/CT revealed thickened mucosa of nasal cavity and ethmoid sinus, which extends to the subcutaneous tissue and part of left orbit, with a maximum standardized uptake value (SUVmax) of 14.25. Several enlarged lymph nodes were also noted in left preauricular, parotid ande cervical regions, with a SUVmax of 14.50.

In October 2019, a 52-year-old male was referred to the otorhinolaryngology outpatient clinic with a complaint of bilateral nasal congestion for two years. Two weeks ago, he developed a fever and swelling of the eyelids. Significant weight loss of 13 kilograms within a three-month period was noted. Physical examination revealed periorbital tenderness and enlarged cervical lymph nodes. Laboratory tests showed markedly elevated levels of C-reactive protein (CRP) and lactate dehydrogenase (LDH). Real time PCR detected positive Epstein-Barr virus (EBV)-DNA. Cranial magnetic resonance imaging (MRI) demonstrated swelling of the left nasal cavity mucosa with inflammation in the paranasal sinus, leading to an endoscopy-guided biopsy (Figures 1A,B). Pathologic examination demonstrated diffuse infiltration of atypical cells with coarse chromatin, prominent nucleoli, and positive staining for cytoplasmic CD3, CD30, CD56, TIA-1 and MUM-1, with Ki-67 index of >40% (Figures 2A–H). These findings confirmed the diagnosis of extranodal ENKTL. Positron emission tomography/computed tomography (PET/CT) showed tumor extension into the nasal cavity, ethmoidal sinus, left orbit, and surrounding lymph nodes (Figure 3). Both MRI and PET-CT did not show any CNS involvement at diagnosis. No evidence of lymphoma infiltration was found in the bone marrow. Therefore, the ENKTL was staged as IIEB with an International Prognostic Index (IPI) score of 2.

The patient underwent sequential chemoradiation, receiving 6 cycles of P-EGEMOX protocol (pegaspargase, etoposide, gemcitabine, oxaliplatin), with the addition of anti-PD1 therapy for the last 4 cycles. This was followed by involved-site radiation therapy (ISRT) with a dose of 50 gray (Gy) at the primary lesion and 45 Gy at corresponding draining lymph nodes. In May 2020, the patient experienced a possible CNS relapse, as indicated by MRI findings (Figures 1C,D), promoting treatment with three cycles of anti-PD1 therapy in combination with high-dose methotrexate (MTX). This was followed by consolidation with autologous stem cell transplantation using a thiotepa-based conditioning regimen.

**Figure 4:**
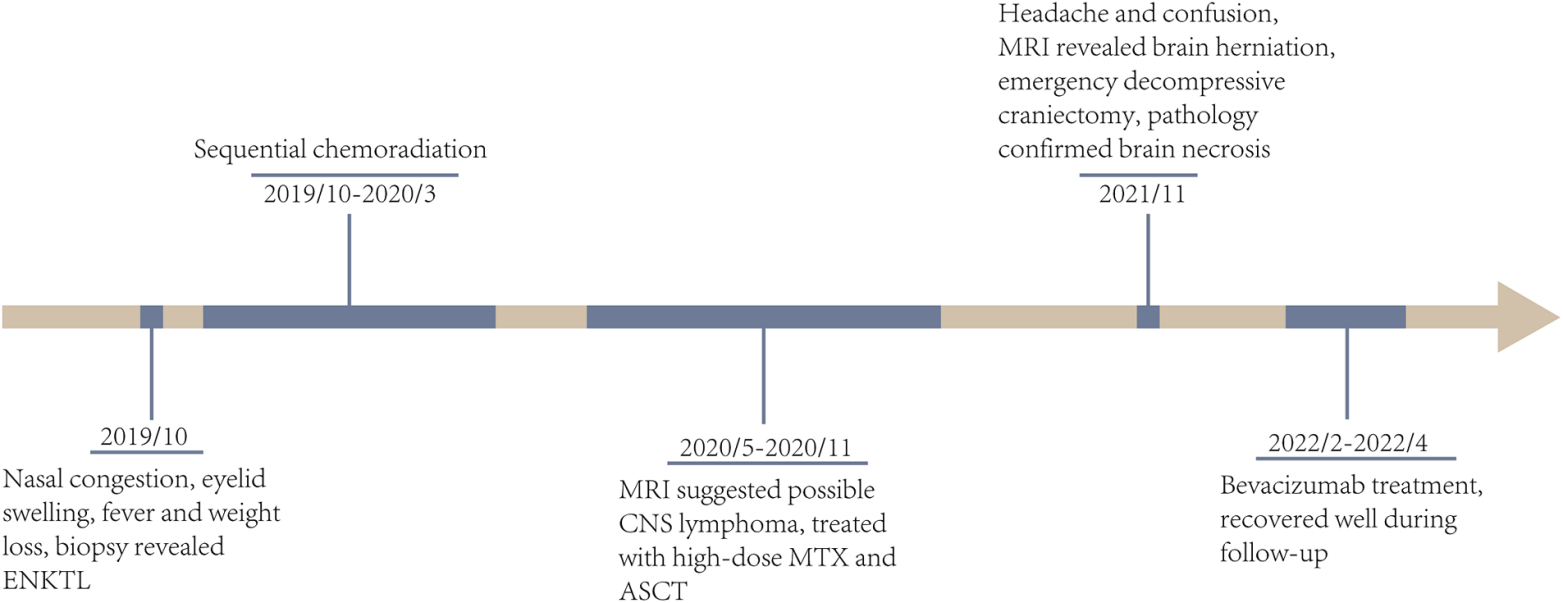
The timeline of clinical courses of the ENKTL patient with brain herniation due to severe radiation necrosis, which were assessed by both MRI and biopsy. ENKTL, extranodal NK/T cell lymphoma; CNS, central niveous system; MTX, methotrexate; ASCT, autologous stem cell transplantation.

In November 2021, the patient was admitted due to symptoms of headache, confusion, and occasional delirium. A brain MRI revealed irregular abnormal lesions in the left frontal lobe with mixed signal intensity on T1-Weighted imaging and heterogeneous high signal on T2- Weighted imaging. Wreath-like enhancement surrounded by lamellar edema, midline shift, and ventricle dilation was observed, suggesting possible lymphoma progression and brain herniation (Figures 1E–H). Emergency decompressive craniectomy was performed, and a pathological examination of the biopsy specimen demonstrated diffuse necrosis of the left frontal lobe without evidence of lymphoma involvement (Figure 2I). Staining for fungi or tuberculosis was negative. CSF examination showed no remarkable findings. Intrathecal dexamethasone was administered, and bevacizumab was given at a dose of 300 mg. The patient gradually recovered and was discharged in good general condition. The timeline of this case was summarized in (Figure 4).

## 3. Discussion

ENKTL has increased risk of CNS involvement because the nasal cavity and paranasal sinus are close to the CNS across bony structures (4). The CNS lymphoma secondary to ENKTL is rare but associated with very poor prognosis. Wu et al. reported that 1.4% of ENKL patients (9 of 662) developed CNS diseases, with a median OS of 9 months and a 1-year OS rate of 44.4% (5). For patients with neurological symptoms, the CSF cytology and biochemistry analysis may help identify leptomeningeal lymphoma infiltration but not parenchymal lesions (6). In case with a history of cranial irradiation, differentiating between lymphoma and radiation necrosis heavily relies on MRI scan. However, this can be challenging as both conditions exhibit similar imaging features, such as a contrast-enhancing mass lesion with central necrosis surrounded by reactive edema. Diffusion-weighted imaging (DWI) can be helpful as lymphoma shows a higher signal on DWI but a lower signal on the apparent diffusion coefficient (ADC) compared to radiation necrosis (7). Nevertheless, when the lesion contains hemorrhage, calcification or fibrous hyperplasia, the mean ADC value decreases, making differentiation difficult (8). Perfusion MRI has high reported diagnostic accuracy as it measures cerebral blood volume (CBV), which reflects tumor angiogenesis; CBV increases in lymphoma, but decreases in brain necrosis (9). Magnetic resonance spectroscopy (MRS) can detect the changes of various metabolites in brain tissue. Tumor progression is associated with elevated choline-creatine ratio and choline-N-acetyl aspartate ratio (10). In necrotic area, a broad peak representing cell debris, including fatty acids and lactate, as well a slight inhibition of some metabolites, could been seen (11).

Initial CNS evaluation is often not performed in most patients unless they exhibit neurological symptoms. CT-guided stereotactic biopsy remains the gold standard for establishing a diagnosis (12). In this case, where the patient required emergency craniotomy for intracranial decompression, and the biopsy confirmed tissue necrosis rather than lymphoma. High-dose corticosteroids are commonly used in neurological patients due to their ability to rapidly alleviate intracranial edema and inflammation; however, if clinically possible, corticosteroids should be avoided prior to biopsy when patients are suspected of having CNS lymphoma because corticosteroids induce transient disease regression that may complicate localization of the biopsy site, and the disappearance of neoplastic cells would obscure pathologic evaluation (13, 14).

P-GEMOX is recommended as one of first-line regimens for advanced ENKTL, and autologous stem-cell transplant as standard consolidation following CR1. Novel agents such as immunodulators are introduced for the treatment of ENKTL (15). After disease remission is achieved, autologous stem-cell transplant may also benefit patients with SCNSL, but the optimial management for SCNSL remains unknown.

Radiation therapy, an important component of initial therapy, can be given in concurrent, sequential or sandwich chemoradiation, and has an essential role in improved overall and disease-free survival, especially in patients with localized ENKTL. Involved-site RT (ISRT) is recommended as the appropriate field as it limits the volume of RT to the region of involvement only. An ISRT dose of 50–55 Gy is recommended when used alone as primary treatment and 45–56 Gy is recommended when used in combination with chemotherapy, which can have deleterious effects on the vasculature of the brain as well as on neuroglial cells and their precursors. Inflammation and blood brain barrier disruption may also indirectly contribute to cellular damage.

Brain tissue necrosis is a delayed form of radiation toxicity, which typically develops one to three years after radiation, although the range is quite broad (16). The pathogenesis of tissue necrosis mainly involves vascular endothelial cell damage as well as disruption of the blood-brain barrier. Tissue necrosis typically occurs at or close to the original tumor site, which has the highest radiation exposure. The clinical course of brain tissue necrosis is highly variable. No causal therapies have been established, and management is primarily symptomatic. The treatment decisions require a balance between the often-competing goals of symptom control and avoidance of side effects. Given the potential role of vascular endothelial growth factor (VEGF) in the pathogenesis, bevacizumab, a monoclonal antibody against VEGF, has been shown activity in alleviating necrosis-associated edema (17). Xu et al. conducted a clinical randomized controlled trial comparing the efficacy of bevacizumab with hormones in radiation necrosis and demonstrated that 38 out of 58 patients (65.5%) in the bevacizumab group were treated effectively, significantly exceed the hormone group (65.5% vs. 31.5%, *P* < 0.001) (18). Currently, bevacizumab is the first-line treatment option for radiation necrosis.

## 4. Conclusion

This case report presents an ENKTL patient who experienced an emergency brain herniation as a result of severe radiation necrosis and highlights the significance of a precise diagnosis in determining the appropriate treatment. Due to their similar symptoms and radiologic features, it is crucial but challenging to distinguish lymphoma from necrosis in practice. Although modern MRI techniques have improved diagnostic performance, stereotactic biopsy is still necessary for a definitive diagnosis when both conditions are indistinguishable on imaging. Even though it is invasive, biopsy should not be postponed in the acute setting, where a sensible treatment plan is essential, especially in patients with complex diseases.

## Data Availability

The raw data supporting the conclusions of this article will be made available by the authors, without undue reservation.
